# Fermentative Bacteria Influence the Competition between Denitrifiers and DNRA Bacteria

**DOI:** 10.3389/fmicb.2017.01684

**Published:** 2017-09-05

**Authors:** Eveline M. van den Berg, Marina P. Elisário, J. Gijs Kuenen, Robbert Kleerebezem, Mark C. M. van Loosdrecht

**Affiliations:** Environmental Biotechnology Group, Department of Biotechnology, Delft University of Technology Delft, Netherlands

**Keywords:** chemostat, denitrification, dissimilatory nitrate reduction, DNRA, Lac/N-ratio

## Abstract

Denitrification and dissimilatory reduction to ammonium (DNRA) are competing nitrate-reduction processes that entail important biogeochemical consequences for nitrogen retention/removal in natural and man-made ecosystems. The nature of the available carbon source and electron donor have been suggested to play an important role on the outcome of this microbial competition. In this study, the influence of lactate as fermentable carbon source on the competition for nitrate was investigated for varying ratios of lactate and nitrate in the influent (Lac/N ratio). The study was conducted in an open chemostat culture, enriched from activated sludge, under strict anoxia. The mechanistic explanation of the conversions observed was based on integration of results from specific batch tests with biomass from the chemostat, molecular analysis of the biomass enriched, and a computational model. At high Lac/N ratio (2.97 mol/mol) both fermentative and respiratory nitrate reduction to ammonium occurred, coupled to partial oxidation of lactate to acetate, and to acetate oxidation respectively. Remaining lactate was fermented to propionate and acetate. At a decreased Lac/N ratio (1.15 mol/mol), the molar percentage of nitrate reduced to ammonium decreased to 58%, even though lactate was supplied in adequate amounts for full ammonification and nitrate remained the growth limiting compound. Data evaluation at this Lac/N ratio suggested conversions were comparable to the higher Lac/N ratio, except for lactate oxidation to acetate that was coupled to denitrification instead of ammonification. Respiratory DNRA on acetate was likely catalyzed by two *Geobacter* species related to *G. luticola* and *G. lovleyi*. Two *Clostridiales* members were likely responsible for lactate fermentation and partial lactate fermentation to acetate coupled to fermentative DNRA. An organism related to *Propionivibrio militaris* was identified as the organism likely responsible for denitrification. The results of this study clearly show that not only the ratio of available substrates, but also the nature of the electron donor influences the outcome of competition between DNRA and denitrification. Apparently, fermentative bacteria are competitive for the electron donor and thereby alter the ratio of available substrates for nitrate reduction.

## Introduction

Nitrate can be reduced by different dissimilatory nitrogen cycle processes. The processes of denitrification and anaerobic ammonium oxidation (anammox) remove nitrogen from the environment by converting nitrate to dinitrogen gas (Kraft et al., 2011). Removal of nitrate is essential to counteract pollutions as a result of anthropogenic N inputs, for example, from wastewaters and brines, prior to its discharge in oceans or rivers (Burgin and Hamilton, 2007; Stein and Klotz, 2016). Alternatively the dissimilatory nitrate reduction to ammonia (DNRA) retains the nitrate-nitrogen in the ecosystem as ammonium and therefore DNRA does not alleviate eutrophication (Jäntti and Hietanen, 2012). Conversion of nitrate to ammonium can also be beneficial, as the ammonium-ion is retained in soils and sediments by absorption, whereas the nitrate anion is easily lost due to leaching (Silver et al., 2001). The DNRA process has received markedly less attention compared to denitrification, and it is the least well-described of the nitrogen cycle processes (Streminska et al., 2012). Although contributions have increased in the past decade, our understanding of the role of DNRA in the environment is limited. As a result, the environmental factors directing the nitrate reduction competition are limitedly understood. To enable control of the nitrate reduction toward the desired end product (N_2_ or ${\text{NH}}_{4}^{+}$), we need to improve this understanding. We focus on the competition between heterotrophic denitrification and DNRA in particular, since autotrophic denitrification and anammox are not considered relevant in organic carbon abundant enrichments.

An environmental factor well-reported to direct the competition between denitrification and DNRA is the C/N ratio of available substrates (Rütting et al., 2011; Kraft et al., 2014). DNRA bacteria have a competitive advantage in nitrate limiting conditions and excess of electron donor, whereas denitrifiers are more competitive when electron donor is limiting (Kraft et al., 2014; van den Berg et al., 2016). This was shown qualitatively in both aquatic and terrestrial environments. Lab cultures provided more insight in the mechanism of this selection by the ratio of available substrates (e.g., Tiedje et al., 1982; Akunna et al., 1994; Yoon et al., 2015; van den Berg et al., 2016). Tiedje et al. (1982) proposed that DNRA could be more favorable under nitrate limiting conditions, because of the capacity of DNRA to accept eight electrons per nitrate, whereas in denitrification five electrons are accepted, even though thermodynamics suggest that the free energy change per nitrate reduced is comparable. van den Berg et al. (2016) studied the effect of available substrates in a continuous enrichment system, using (non-fermentable) acetate and nitrate as substrates at variable acetate concentrations in the influent to alter the electron donor and acceptor in the influent (i.e., Ac/N ratio). For a wide range of substrate ratios, a steady state was established where denitrification and DNRA coexisted, and both acetate and nitrate were limiting. A model showed that this behavior could be attributed to the differences in the process stoichiometries of DNRA and denitrification, i.e., use of acetate per nitrate in the metabolism (van den Berg et al., 2016).

Ecological niches allowing DNRA to occur have typical an excess of carbon substrate, this will also give the possibility for fermentative bacteria to be active at the same time. Fermentative conversions will have an influence on the type of carbon source available for nitrate reduction and thereby potentially affect the relative occurrence of DNRA and denitrification. Differences in use of electron donors for the reduction of nitrate are limitedly understood. For some pure cultures, yields have been reported for different substrates (e.g., Strohm et al., 2007). Rehr and Klemme (1989) studied denitrifying and DNRA pure cultures competing for nitrate using lactate and different additional amounts of glucose in a chemostat system (Rehr and Klemme, 1989). They suggest the bacterium performing fermentation and DNRA, as opposed to denitrification only, had a competitive advantage, because it could obtain energy from both fermentation and electron donor oxidation coupled to acetate production. Akunna et al. (1993) reported batch cultivations with sludge from an anaerobic digester and showed that nitrate reduction to ammonia occurs only for the fermentable substrates glucose and glycerol, but not for lactate, acetate and methanol. These observations suggest that DNRA can be more competitive when the organic electron donors available are more reduced, because of additional occurrence of fermentative DNRA. This “fermentative DNRA” is bioenergetically advantageous compared to pure fermentation, because DNRA allows more acetate production. Hence, using fermentative DNRA more substrate level ATP can be produced from acetyl-CoA, without compromising the required redox balance, as the reduction equivalents are channeled off to reduce nitrate (Cole and Brown, 1980; Polcyn and Podeszwa, 2009; Kraft et al., 2011). In fermentative DNRA, the electrogenic yield of the nitrate reduction can be absent or lower compared to the respiratory DNRA, and varies for different conditions (de Vries et al., 1982; Pope and Cole, 1982; Cole, 1996; Otte et al., 1999).

Previous we reported on the effect of C/N ratio with a non-fermentable substrate (van den Berg et al., 2016), and in the present study we have extended the complexity by using a fermentable substrate to test the influence of fermentative conversions on the competition between DNRA and denitrification. Lactate was chosen as a “model”-fermentable energy—and C-source, as for this substrate fermentation pathway options are relatively limited compared to carbohydrates like glucose, thereby minimizing the additional complexity of the system. We hypothesized that lactate fermentation only occurs when nitrate is depleted, as observed previously in acetate—nitrate studies (van den Berg et al., 2016) and that C/N effect will have the same stoichiometric basis.

We studied the effect of lactate/N (Lac/N) in a continuous enrichment (i.e., mixed) culture grown on mineral medium with lactate as electron donor and nitrate as electron acceptor. The reactor was operated at a low enough dilution rate to allow growth of both the denitrifying and fermentative and respiratory DNRA bacteria (Cole and Brown, 1980; Kraft et al., 2014; van den Berg et al., 2015). Lactate concentrations were adapted to create different ratios of lactate per nitrate (Lac/N ratio) in the influent, comparable in terms of electron equivalents to the acetate/N (Ac/N) ratios used in our previous study (Table 1) (van den Berg et al., 2016). As the additional complexity in the system, compared to acetate use, obscured direct interpretation of the conversions, batch tests were performed with the steady state cultures to identify the potential capacities for pathways of the relevant *e*-donors, and *e*-acceptors involved in the steady states. In addition, a model was developed to evaluate the possible pathway contributions in the overall conversions. Furthermore, the steady state microbial communities were analyzed using amplicon sequencing, verified by denaturing gradient gel electrophoresis (DGGE), and fluorescent *in situ* hybridization (FISH).

**Table 1:** Lactate/nitrate influent ratios translated to C/N ratios.

**Day**	**Lac/N (mol/mol)**	**C/N (C-mol/N-mol)**	**Comparable Ac/N (mol/mol)**
0–45	2.97	8.92	4.46
46–110	1.15	3.45	1.87
111–135	0.63	1.88	0.94

*As the results are compared with acetate influent, in the third column the influent acetate/N ratio representing the same amount of influent electron equivalents as the Lac/N is listed. Lactate can donate 12 electrons and acetate eight, so they both donate four electrons per C-mol*.

## Materials and methods

### Chemostat operation

Continuous culture experiments were performed in an anoxic chemostat reactor, a double-jacket glass reactor with a working volume of 2 l (Applikon, Delft, The Netherlands). The bioreactor was inoculated with a sample of 2 l of activated sludge (3–3.5 g dry matter/l) from the Wastewater Treatment Plant Harnaschpolder (Delft, The Netherlands). The reactor was operated in anoxic conditions by sparging a constant flow of 100 ml min^−1^ of nitrogen gas, by means of a mass flow controller (Brooks Instrument, The Netherlands). The stirring speed was kept at 400 rpm, a stirrer with two standard geometry blades was used. The pH of the culture was monitored by a pH electrode (Mettler Toledo, USA) and controlled to a set point of 7.1 ± 0.05 with 0.5 M NaOH and 0.5 M HCl by a pH biocontroller, ADI 1030 (Applikon, Delft, The Netherlands). To monitor acid and base consumption the respective bottles were periodically weighted. The redox potential of the culture was monitored by a redox electrode (Mettler Toledo, USA). Data acquisition of online measurements (redox potential, pH, acid, and base dosage) was accomplished by MFCS/win (Sartorius Stedium Systems, USA). A water jacket and cryostat bath (Lauda, Germany) was used to maintain the reactor temperature at 22°.

Peristaltic pumps (Masterflex®, USA) were used to supply influent and remove effluent, controlling the dilution rate of the system to 0.027 ± 0.001 h^−1^. The effluent pump was controlled by a volume level sensor. The influent pump was calibrated to deliver two separate medium flows at equal rates to a total constant rate of 53 ml h^−1^, which corresponds to the mentioned dilution rate. Both culture media were autoclaved before use and sparged with a small flow of nitrogen gas while connected to the chemostat to ensure anaerobic conditions. The pump tubing was Noroprene Masterflex®, all other tubing was Noroprene. The substrate medium (A) contained lactate prepared from a sodium DL-lactate solution syrup, 60 % (w/w) to obtain a concentration of 35.0 mM for Lac/N 2.97, 13.5 mM for Lac/N 1.15, and 7.38 mM for Lac/N 0.63 (Table 1). The mineral medium (B) contained per liter: 23.5 mmol NaNO_3_ as nitrogen source and electron acceptor, 22.0 mmol KH_2_PO_4_, 1.2 mmol MgSO_4_.7H_2_O, 1.5 mmol NaOH, 1.5 mg yeast extract and 5 ml trace element solution (Vishniac and Santer, 1957), with the ZnSO_4_.7H_2_O concentration reduced to 2.2 g per liter and use of sodium molybdate instead of ammonium molybdate. In a parallel reactor with identical set up the Lac/N 2.97 mol/mol steady state and Lac/N 1.15 steady state were reestablished from the chemostat culture effluent (2 l) from another steady state, Lac/N 1.15 and Lac/N 0.63 respectively, and, in addition, 10 ml of fresh activated sludge.

### Batch experiments

One liter of chemostat effluent was collected on an ice bath under anoxic conditions by continuously flushing with a low flow of dinitrogen gas. Prior to the batch tests the biomass concentration was determined as volatile suspended solids (VSS). The effluent was centrifuged during 20 min at 10,000 rpm and 4°C. The cell pellet was resuspended in a phosphate buffer (26.8 mM, pH 7.00), which was flushed with nitrogen gas for 30 min to minimize dissolved oxygen concentration. The batch experiments were performed in 20 or 30 ml serum bottles equipped with rubber stoppers and aluminum cap sealers. Batch tests were not duplicated. The carbon source and electron acceptor were added in varying combinations according to Tables 2, 3. Initial concentrations of electron donor were always 5 mM and electron acceptor 4 mM, in a batch volume of 10 or 20 ml. To estimate production of dinitrogen gas in the batch tests, in particular cases the cells were additionally incubated with 10% acetylene in the gas phase to block the nitrous oxide reductase. The observed N_2_O production in these batch tests is an indication of the denitrifying potential of the biomass. The bottles were sequentially sealed, flushed with dinitrogen gas with a syringe tip through the rubber lid and submitted to vacuum to release dissolved gasses. During the experiments a slightly positive pressure was maintained in the vials to avoid oxygen leakage into the bottles and to facilitate sampling. Incubation times varied between 3 and 6 h and the sampling interval varied from 45–90 min (Figures S1, S2).

**Table 2:** List of batch tests performed in the culture of Lac/N ratio of 2.97 and respective combination of electron donor and acceptor.

**Test**	**Electron donor**	**Electron acceptor**
A	Lactate	–
B	Lactate	Nitrate
C	Lactate	Nitrite
D	Acetate	Nitrate
E	Acetate	Nitrite
F	Propionate	Nitrate
G	Propionate	Nitrite

*Initial concentrations of electron donor were always 5 mM and electron acceptor 4 mM, in a batch volume of 10 ml*.

**Table 3:** List of batch tests performed in the culture of Lac/N ratio of 1.15 and respective combination of electron donor and acceptor.

**Test**	**Electron donor**	**Electron acceptor**	**Acetylene concentration [% (v/v)]**
H	Lactate	–	–
I	Lactate	Nitrate	–
J	Lactate	Nitrite	–
K	Acetate	Nitrate	–
L	Acetate	Nitrite	–
M	Propionate	Nitrate	–
N	Propionate	Nitrite	–
O	Lactate	Nitrate	5
P	Acetate	Nitrate	5
Q	Propionate	Nitrate	5

*Initial concentrations of electron donor were always 5 mM and electron acceptor 4 mM, in a batch volume of 20 ml*.

### Analytical methods

Either for chemostat or batch experiments, periodic samples were taken, respectively, from the reactor or vials and centrifuged for 4 min at 13,000 rpm. The supernatant was collected to measure nitrogen compounds (ammonium, nitrite, and nitrate), lactate and volatile fatty acids concentrations. Test strips (Merck Millipore, Germany) were used to test qualitatively the presence of nitrate and nitrate. Ammonium concentrations were quantified spectrophotometrically with a commercial cuvette test kit (Hach Lange, Germany), with a lower detection limit of 1 μM. In case of a test strip positive result nitrate, and nitrite were tested with a similar test kit, with lower detection limits of respectively 0.02 and 0.01 mM. Lactate and volatile fatty acids, such as acetate and propionate, were determined with high-performance liquid chromatograph using a BioRad Animex HPX-87H column. Together with the set of samples, standards for acetate and lactate (5 mM) and propionate (5 and 18 mM) were analyzed in HPLC to validate the calibration curve and results. Oxygen, carbon dioxide, nitric oxide, and nitrous oxide partial pressure in the off-gas of the chemostat were monitored using a gas analyser (NGA 2000, Rosemount, USA). The gas flow through the reactor of 100 ml min^−1^ was chosen to maintain sufficient flow through the gas analyzer (80 ml min^−1^). Nitrous oxide partial pressure in the headspace of the batch vials was measured off-line on an Agilent 6890 Gas Chromatograph, with reported protocol (Kampschreur et al., 2008). The system was considered to be in steady state when conversion rates were stable for at least 7 days, i.e., five volume changes.

Biomass concentrations of the chemostat culture were measured by determination of the volatile suspended solids (VSS) concentrations using reported methods for DNRA bacteria (van den Berg et al., 2015). To determine the biomass concentration, the reactor effluent was centrifuged (10,000 rpm for 20 min) and the pellet was dried at 105°C. Subsequently the ash content was subtracted to obtain VSS concentration. The ash content was determined by burning the organic parts of the dried pellet at 550°C. Protein concentrations were measured by the bicinchoninic acid method using BC Assay Protein Quantification Kit (Interchim, France) following manufacturer's instructions.

A balance of degree of reduction and a charge balance of incoming and exiting elements in the chemostat were set up to verify the consistency of our measurements. The concentration of volatile suspended solids (VSS) was used as biomass concentration. The system has a relatively high dilution rates, compared to e.g., soils. As a result biomass decay is not significant and immobilization/re-mineralization negligible. Hence, ammonium production was attributed to nitrate reduction by DNRA. As emissions of nitric and nitrous oxide were not detected, the nitrogen not accounted for in ammonium, nitrate, nitrite or biomass was assumed to be converted to N_2_. Sulfide was not detectable with the methylene blue method (Cline, 1969), with a lower limit of 1 μM. To calculate the concentration of bicarbonate species in the chemostat solution, the electro-neutrality equation for the charged species in the chemostat was solved with pKa values listed in Table S1. In the batch conversions, the end product concentrations are used to calculate percentages of N-conversion as a percentage of the consumed nitrate or nitrite. In the batches with acetylene, the N_2_ production was estimated from the end product concentration of nitrous oxide, subtracted by the nitrous oxide produced in the batch without acetylene.

### DGGE and amplicon sequence analysis of PCR amplified 16S genes

The microbial community structure of the culture was analyzed by amplicon sequence analysis. To verify the results, the DNA extracts were additionally analyzed using denaturing gradient gel electrophoresis (DGGE), as in both methods a different PCR protocol is applied. Biomass samples were collected from the reactor, and centrifuged and stored at −20°C. The sample 2.97 a was taken on day 36, 1.15 a on day 107, 0.63 on day 134, 2.97 b on day 40 of the parallel reactor and 1.15 b on day 25 after restarting the parallel reactor. The genomic DNA was extracted using the UltraClean Microbial DNA isolation kit (MO BIO, Carlsbad, CA, USA), following manufacturer's instructions. The extracted DNA products were evaluated on 1% (w/v) agarose gel.

In amplicon sequencing the extracted DNA was processed by Novogene Bioinformatics Technology (Beijing, China). Amplification of part of 16S rRNA gene was performed using a paired-end Illumina HiSeq platform to generate 450 bp pair-end reads (Raw PE), which were trimmed to 250 bp. Amplicons were generated targeting hypervariable regions (V3-4) of 16S rRNA genes using specific primers (341F-806R) with the barcode. All PCR reactions were carried out with Phusion® High-Fidelity PCR Master Mix (New England Biolabs). Quantification and qualification of the PCR products was done by electrophoresis on a 2% agarose gel. PCR products were mixed in equidensity ratios and then purified with Qiagen Gel Extraction Kit (Qiagen, Germany). Sequencing libraries were generated using TruSeq® DNA PCR-Free Sample Preparation Kit (Illumina, USA) following manufacturer's recommendations and index codes were added. The library quality was assessed on the Qubit@ 2.0 Fluorometer (Thermo Scientific) and Agilent Bioanalyzer 2100 system. The library was sequenced on an IlluminaHiSeq2500 platform and 250 bp paired-end reads were generated (Table S2). The data was split by assigning pair-end reads to samples based on their unique barcode and truncated by cutting off the barcode and primer sequence. The paired-end reads were merged using FLASH (V1.2.7), and the splicing sequences were called raw tags. Quality filtering on the raw tags was performed under specific filtering conditions to obtain the high-quality clean tags according to the Qiime (V1.7.0) quality controlled process. Chimeras were detected by comparing with the reference database (Gold database) using UCHIME algorithm (UCHIME Algorithm) and subsequently removed to obtain “Effective Tags”. Sequences analysis were performed by Uparse software (Uparse v7.0.1001). Sequences with ≥97% similarity were assigned to the same consensus sequences. The consensus sequences were classified using the Greengene Database, based on the RDP classifier algorithm (Version 2.2). Alpha diversities were calculated for the different consensus sequence abundances (Table S3), which were normalized using a standard of sequence number corresponding to the sample with the least sequences, were used.

In DGGE analysis the extracted DNA was used as for PCR amplification of the 16S rRNA gene. The set of primers used was the 341F (containing a 40-bp GC clamp) and 907R (Schäfer and Muyzer, 2001). The used PCR thermal profile started with a pre-cooling phase at 4°C for 1 min, followed by initial denaturation at 95°C for 5 min, 32 cycles of 95°C for 30 s, 55°C for 40 s, 72°C for 40 s, followed by an additional extension step at 72°C for 30 min. DGGE band isolation and DNA sequencing were performed as described by Bassin et al. (2012) for 16S rRNA. The obtained 16S rRNA gene sequences were manually corrected using the program Chromas Lite 2.1.1 (http://technelysium.com.au.) The corrected sequences bands and consensus sequences from amplicon sequencing analysis were compared with those stored in GenBank using the Basic Local Alignment Search Tool algorithm (http://www.ncbi.nlm.nih.gov/blast.) The sequences have been deposited in the GenBank under accession numbers MF445187-MF445194 and MF445197-MF445207.

### Fish and microscopic analysis of the culture

Fluorescent *in situ* hybridization (FISH) was performed as described by Johnson et al. (2009), using a hybridization buffer containing 35% (v/v) formamide. The applied probes are listed in Table 4. The general probe mixture EUB338 labeled with Cy5 was used to indicate all eubacteria species in the sample. In the shown result, we used combined this probe with either the Beta42a probe, labeled with Cy3 (plus an unlabeled Gamma42a probe, to minimize erroneous hybridizations of Beta42a) or a mixture of two Cy3 labeled probes specifically designed for the detection of the 16S rRNA of the enriched *Geobacter* related microorganisms (GeoBac464 and GeoBacII464). The specificity for the GeoBacII464 probe, presented in this study, is included in Table S4. Probes were synthesized and 5′ labeled with either the FLUOS or with one of the sulfoindocyanine dyes Cy3 and Cy5 (Thermo Hybaid Interactiva, Ulm, Germany). Slides were observed with an epifluorescence microscope (Axioplan 2, Zeiss, Sliedrecht, The Netherlands), and images were acquired with a Zeiss MRM camera and compiled with the Zeiss microscopy image acquisition software (AxioVision version 4.7, Zeiss) and exported as TIFF format.

**Table 4:** Probes used in the FISH analysis.

**Probe**	**Sequence**	**Dye**	**Specificity**	**Reference**
EUB338mix	gcwgccwcccgtaggwgt	Cy5	Most bacteria	Amann et al., 1990; Daims et al., 1999
Beta42a	gccttcccacttcgttt	Cy3	*Betaproteobacteria*	Manz et al., 1992
GeoBac464	agcctctctacacttcgtc	Cy3	*Geobacter* ribotype	van den Berg et al., 2015
GeoBacII464	aacctccgtacacttcgcc	Cy3	*Geobacter* ribotype	This study

### Model

A simple model was used to deduce the contributions of different possible conversions for the reactor steady states. First, for each pathway considered the conversion stoichiometry was established. The lactate fermentation stoichiometry (Equation 1) was based on thermodynamic state analysis as described by Kleerebezem and Van Loosdrecht (2010) combined with the Gibbs energy dissipation concept proposed by Heijnen and Van Dijken (1992) and Heijnen et al. (1992) to estimate biomass yields and use of Gibbs energies of formation as established by Thauer et al. (1977). The resulting biomass yield was similar to measured yields for lactate fermentation by Seeliger et al. (2002).

(1) $$\begin{array}{l} -8.88Lac^{-}-0.20NO_{3}^{-}-0.53H^{+}+1.00CH_{1.8}O_{0.5}N_{0.2}\\ +\;3.15Ac^{-}+5.40Prop^{-}+3.15CO_{2}+3.35H_{2}O \end{array}$$

In Equation (1) the abbreviation *Lac* is used for lactate, *Ac* for acetate and *Prop* for propionate. Assuming that in fermentative DNRA using lactate the same amount of ATP is harvested per acetate produced as in lactate fermentation, and nitrate reduction is not electrogenic, the stoichiometry was written as Equation (2).

(2) $$\begin{array}{l} -3.51Lac^{-}-1.55NO_{3}^{-}-3.24H^{+}+1.00CH_{1.8}O_{0.5}N_{0.2}\\ +3.15Ac^{-}+1.33NH_{4}^{+}+3.15CO_{2}+2.03H_{2}O \end{array}$$

The measured metabolic stoichiometry for respiratory DNRA using acetate described by van den Berg et al. (2016) was used (Equation 3), as a very similar experimental setup was used in that study.

(3) $$\begin{array}{l} -1.96Ac^{-}-1.44NO_{3}^{-}-4.63H^{+}+1.00CH_{1.8}O_{0.5}N_{0.2}\\ +\;1.24NH_{4}^{+}+2.92CO_{2}+1.89H_{2}O \end{array}$$

For the stoichiometry of denitrification coupled to partial lactate oxidation, the result of thermodynamic calculations was combined with the described yield per mole nitrate for denitrification using acetate in a similar system (van den Berg et al., 2016), as there is a discrepancy between theoretical and practical energy gain (Strohm et al., 2007; van den Berg et al., 2015). The yield per mol nitrate was assumed comparable for partial lactate oxidation to acetate and acetate oxidation, because these processes have a similar ATP yield per electron mole. This resulted in a stoichiometry shown in Equation (4).

(4) $$\begin{array}{l} -3.44Lac^{-}-2.33NO_{3}^{-}-2.66H^{+}+1.00CH_{1.8}O_{0.5}N_{0.2}\\ +\;3.11Ac^{-}+1.06N_{2}+3.11CO_{2}+4.37H_{2}O \end{array}$$

In the model, the contribution of the individual reaction rates to the overall reaction observed was estimated using an optimization procedure. The differences between the computed and the measured rates per compound were weighted by a factor equal to the inverse of the standard deviation of the compound measurements. Subsequently, the computed sum of the squared errors was minimized to obtain the optimal pathway contributions to describe the data. Biomass fractions for the contributing processes were extracted from this result by taking the separate computed biomass production rates and dividing this by the summed biomass production rate. Note that the biomass yield in fermentative DNRA would be higher, when the nitrate reduction was electrogenic. However, this would hardly affect the model outcome, as biomass was a less important parameter in the evaluation due to the relatively high standard deviation.

## Results

### Chemostat operation

To explore the C/N effect in the competition between denitrification and DNRA with lactate as carbon source and electron donor and nitrate as *e*-acceptor and N source, a chemostat system was inoculated with activated sludge as mixed microbial community. Initially, the supplied Lac/N ratio was 2.97 mol/mol, which was subsequently decreased to 1.15 and 0.63 mol/mol. The system was considered in steady state when conversions were stable for at least five retention times. In all steady states, nitrate concentrations were always below the detection limit (<0.02 mM) and therefore considered limiting. Furthermore, no nitrite was detected in the culture (<0.01 mM), neither were nitric oxide or nitrous oxide in the off-gas (both detection limits of 5 ppm). Steady state conversions and balances are shown in Table 5. For all steady states, the protein content of the biomass was 0.59 ± 0.03 mg protein/mg VSS and the redox potential was −380 ± 50 mV.

**Table 5A:** Net conversion rates (mmol/h) in the reactor steady states for the different influent Lac/N ratios (mol/mol).

**Lac/N**	**Lac^−^**	**${\text{NO}}_{3}^{-}$**	**H^+^**	**Biomass**	**Ac^−^**	**Prop^−^**	**${\text{NH}}_{4}^{+}$**	**CO_2_**
2.97	–1.77 ± 0.06	–0.59 ± 0.02	–1.78 ± 0.02	0.69 ± 0.05	0.32 ± 0.02	0.76 ± 0.03	0.41 ± 0.02	1.87 ± 0.07
1.15	–0.69 ± 0.02	–0.60 ± 0.02	–1.55 ± 0.02	0.54 ± 0.04	0	0.07 ± 0.00	0.23 ± 0.01	1.24 ± 0.06
0.63	–0.37 ± 0.06	–0.59 ± 0.02	n.d.	n.d.	0	0	0	n.d.

*Calculations for the bicarbonate concentration are included in the Supplementary Materials. n.d., Not determined*.

**Table 5B:** Balance residuals (%) for the conversions in the reactor steady states calculated from the conversion rates.

**Lac/N**	**Carbon**	**Charge**	**Degree of reduction**
2.97	3	12	2
1.15	4	16	11

*As no biomass, carbon dioxide and proton consumption measurements were available for the steady state receiving 0.63 Lac/N, balances could not be evaluated for this culture*.

Starting from activated sludge, a culture was enriched and grown at a high influent Lac/N ratio of 2.97 mol/mol. The high excess of the electron donor lactate enabled the possibility for nitrate reduction using partial lactate oxidation to acetate. In the obtained steady state, 23% of the nitrate was incorporated into biomass and 69% of the nitrate was reduced to ammonium, which was attributed to DNRA activity. No residual lactate was observed and the culture contained significant amounts of the lactate fermentation products acetate and propionate. ~18% of the lactate was converted to acetate and 42% was converted to propionate. The biomass yield in this culture was 9.6 ± 0.5 g VSS/mol lactate.

Subsequently, the excess influent lactate was reduced (but still in excess) to achieve the Lac/N ratio of 1.15. In this case only 10% of lactate was converted to propionate and there was no residual acetate. 58% of influent nitrate was converted to ammonium, and 20% was used for biomass production. The part of converted nitrate unaccounted for, 38%, was assumed to be converted to dinitrogen gas, with an estimated rate of 0.13 ± 0.02 mmol/h. In addition, acid consumption was lower as less acid was consumed in the nitrate reduction to dinitrogen gas as compared to DNRA (Table 5). For this decreased Lac/N ratio of 1.15, the yield on lactate was increased to 19.2 ± 1.0 g VSS/mol lactate, since a larger fraction of lactate was respired.

To validate conversions at dual limitation of electron donor and ${\text{NO}}_{3}^{-}$-N, the influent Lac/N ratio was further decreased to 0.63 mol/mol. At this steady state, all influent nitrate was denitrified, apart from assimilation, and the effluent contained no residual lactate or fermentation products. Hence, both the DNRA and fermentative bacteria were outcompeted and only denitrification remained.

### Batch experiments

To estimate the possible catabolic processes occurring in the reactor steady states, simultaneous batch tests were performed using resting cell suspension obtained from the steady state reactor biomass. Additionally, the consumption rates of different substrates were evaluated. Different combinations of carbon sources and electron acceptors were tested. Lactate, acetate and propionate were tested separately as electron donors since these were available in the different steady states of the chemostat experiment. Carbon sources were always supplied in higher initial concentrations (5 mM) than electron acceptor (4 mM) to assure electron-excessive conditions. A full overview of the batch results can be found in Figures S1, S2 and the resulting conversion rates in Tables S5, S6.

**Figure 1:**
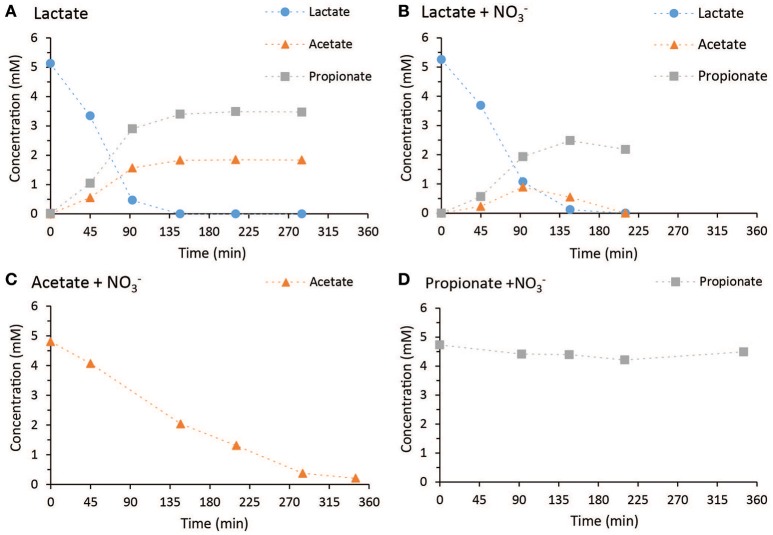
The concentration profiles of the batch tests performed with the culture operated at Lac/N ratio 2.97. The tested substrate combinations shown are **(A)** lactate in the absence of an electron acceptor, **(B)** lactate with nitrate, **(C)** acetate with nitrate and **(D)** propionate with nitrate.

In Figure 1 the concentration profiles of the four most relevant the batch tests performed with the culture operated at Lac/N ratio 2.97 are shown. In the absence of an electron acceptor, one mole lactate was fermented to 0.37 mole of acetate and 0.69 mole of propionate (Figure 1A), with no measurable production of H_2_. Batch tests using lactate together with nitrate or nitrite as electron acceptor showed similar rate of propionate production and a transient acetate accumulation (Figure 1B). The acetate accumulation was lower with nitrite as electron acceptor compared to nitrate. For both electron acceptors, lactate and acetate were consumed simultaneously and the production rate of propionate was lower than in the absence of an electron acceptor.

With acetate as electron donor, the conversion appeared to be slower than lactate depletion for the same concentrations of respective electron acceptor (Figure 1C). Propionate, when used as an electron donor, was only consumed for the conversion of nitrate into nitrite but at an insignificant rate (Figure 1D).

In the batch tests with the cells from the chemostat culture operated at Lac/N ratio 1.15, when only 58%-N was converted to ammonium, the lactate fermentation stoichiometry observed was similar to the high Lac/N culture. Also, the lactate consumption rates were similar, both for using nitrate as electron acceptor and for pure fermentation. However, consumption of lactate was slower when nitrite was used as electron acceptor instead of nitrate. The propionate consumption rate with nitrate as electron acceptor was much higher compared to the Lac/N 2.97 culture. However, no propionate conversion was observed with nitrite. In the incubations with acetate, the relative amounts of ammonium and nitrous oxide produced were slightly higher for use of nitrite than nitrate.

Overall only 3–17% of the converted nitrogen could be recovered as ammonium or nitrous oxide. To estimate the production of dinitrogen gas, in additional batch tests, cells were incubated with acetylene in the gas phase (Table S6). These tests were performed with the nitrate incubations only and showed a great denitrifying potential, as 58–84% of the nitrate was converted to nitrous oxide.

### Microbial population

**Figure 2:**
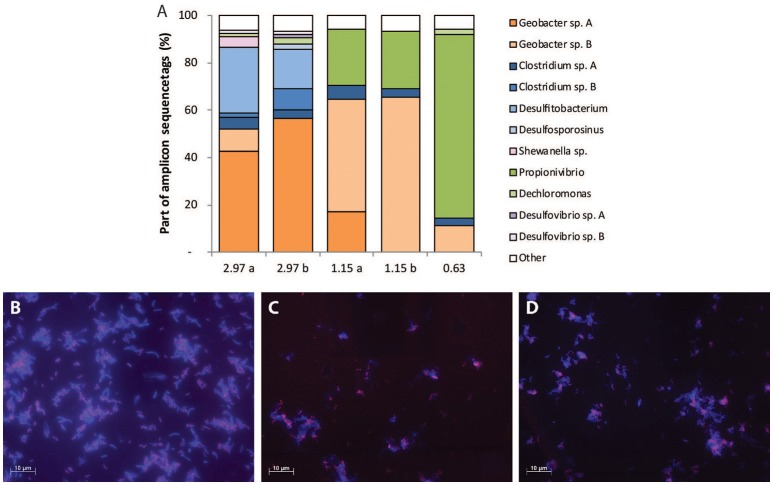
**(A)** Amplicon sequencing results, including consensus sequences which make up ≥1% of amplicon sequences. For the steady states of ratio 2.97 and 1.15 two samples were analyzed. **(B)** FISH micrograph of the steady state population receiving 2.97 Lac/N influent. **(C,D)** FISH micrograph of the steady state population receiving 1.15 Lac/N influent. In **(B–D)** the cells were stained with Cy5-labeled probes for bacteria (EUB338mix, blue), and was in **(B,C)** combined with Cy3-labeled probes specific for the *Geobacter* species (GeoBac464 and GeoBacII464). There, cells colored purple indicate cells to which the probes EUB338mix, and GeoBac464 or GeoBacII464 were hybridized. Whereas, in **(D)** Cy3-labeled probes for *Betaproteobacteria* (Beta42a) were used and cells colored purple indicate cells to which the probes EUB338mix and Beta42a were hybridized.

The microbial community structure for the different chemostat steady states was analyzed by amplicon sequencing, and additional DGGE for verification, and fluorescent *in situ* hybridization (FISH) (Figure 2). The consensus sequences (250 bp) which were made up of ≥1% of amplicon sequences were analyzed using BLASTn. Alpha diversities for the different samples are included in Table S3. Samples indicated with “b” (Figure 2, 2.97 b and 1.15 b) were taken from the reestablished steady states. For the steady state at high Lac/N ratio of 2.97, where fermentation of lactate and DNRA were the main conversions, three predominant consensus sequences were observed in the amplicon result (Figure 2, 2.97 a and 2.97 b). On the basis of a limited identification with the 250 bp only, two of the dominant taxa were a member of the genus *Clostridium*, another related to a *Desulfitobacterium* species (blue, Figure 2), both described to be capable of fermentation of lactate. The other two dominant consensus sequences related most closely to the *Geobacter* species (orange, Figure 2), both related 100% to a ribotype identified in a DNRA enrichment culture (van den Berg et al., 2016). One was identical to the *G. luticola* related (97%) ribotype (sp. A in Figure 2) and the other was identical to the *G. lovleyi* related (97%) ribotype (sp. B in Figure 2).

When the Lac/N ratio was reduced to 1.15 and fermentation, denitrification and DNRA appeared to co-exist, the two *Geobacter* ribotypes remained dominant in the chemostat and, in addition, one of the *Clostridium* species (A) remained present (Figure 2A). Furthermore, a consensus sequence for bacteria closely related to *Propionivibrio militaris* was present (green, Figure 2A), which was assumed to be responsible for the denitrification (Thrash et al., 2010).

In the culture receiving the influent Lac/N of 0.63, only denitrification was observed and the presumed denitrifier consensus sequence, closely related to *P. militaris*, was dominant in the population (Figure 2A). Also the *Geobacter* sp. A and *Clostridium* sp. A consensus sequence were detected in this culture. For each of the samples, similar results were obtained using DGGE profiling (Figure S3).

To verify the results of the amplicon analysis and to estimate the relative abundance of predominant organisms, the populations of the steady states with Lac/N 2.97 and 1.15 were analyzed using FISH (Figure 2B). The relative abundance of the *Geobacter* population was analyzed using a combination of the very specific FISH probes developed for each of both *Geobacter* ribotypes (van den Berg et al., 2016, Table S4). As *P. militaris* belongs to the *Betaproteobacteria*, and was the only dominant *Betaproteobacterium* observed in the sequencing analysis, its relative abundance was assumed to be covered by FISH probes for *Betaproteobacteria*. The *Clostridiales* and *Desulfitobacterium* members found in amplicon sequencing were not targeted with a (group-) specific probe and therefore largely made up the population only hybridizing with the probe for eubacteria (blue colored cells, Figure 2B). A FISH probe for *Gammaproteobacteria* was used to determine the relative abundance of the *Shewanella* species observed in Figure 2, 2.97 a, but no hybridization was observed (not shown).

For the steady state with Lac/N ratio 2.97, about half of the cells hybridized with the specific probes for the *Geobacter* species, colored in purple in the FISH image (Figure 2B), and therefore identified as the *Geobacter* related biomass. Separate probing of the two species is included in the Supplementary Materials. The remaining cells, colored in blue, were assumed to belong the consensus sequences of the *Clostridiales*, and there was no signal of *Betaproteobacteria*. In the population of the steady state with Lac/N ratio 1.15 (Figures 2C,D), the relative abundance of the two combined *Geobacter* species remained. However, a significant part of the population was then identified as *Betaproteobacteria*, most likely of the consensus sequence related to *P. militaris*.

### Model based evaluation

The contribution of the different metabolic pathways to the overall conversion in the system was estimated using the mixed culture model proposed in the materials and methods section. The error between measured and calculated residual concentrations in the system was minimized by optimizing the contribution of the individual pathways proposed. The following assumptions were used in defining the different metabolic pathways occurring:

- Propionate consumption was negligible in the batch tests, therefore the residual propionate concentration was used as a measure for lactate fermentation in the system.
- Ammonium production (including assimilatory consumption) in the system was attributed to DNRA. Respiratory DNRA was assumed to be coupled to acetate oxidation, because the presumed DNRA bacteria were the *Geobacter* species observed in the microbial community. Both species were tested for their capacity to convert lactate. A *Geobacter* sp. B enrichment grown on acetate (unpublished data) showed no activity on lactate. The *Geobacter* sp. A has been isolated as pure culture (manuscript in preparation) and was not able to grow on lactate.

**Figure 3:**
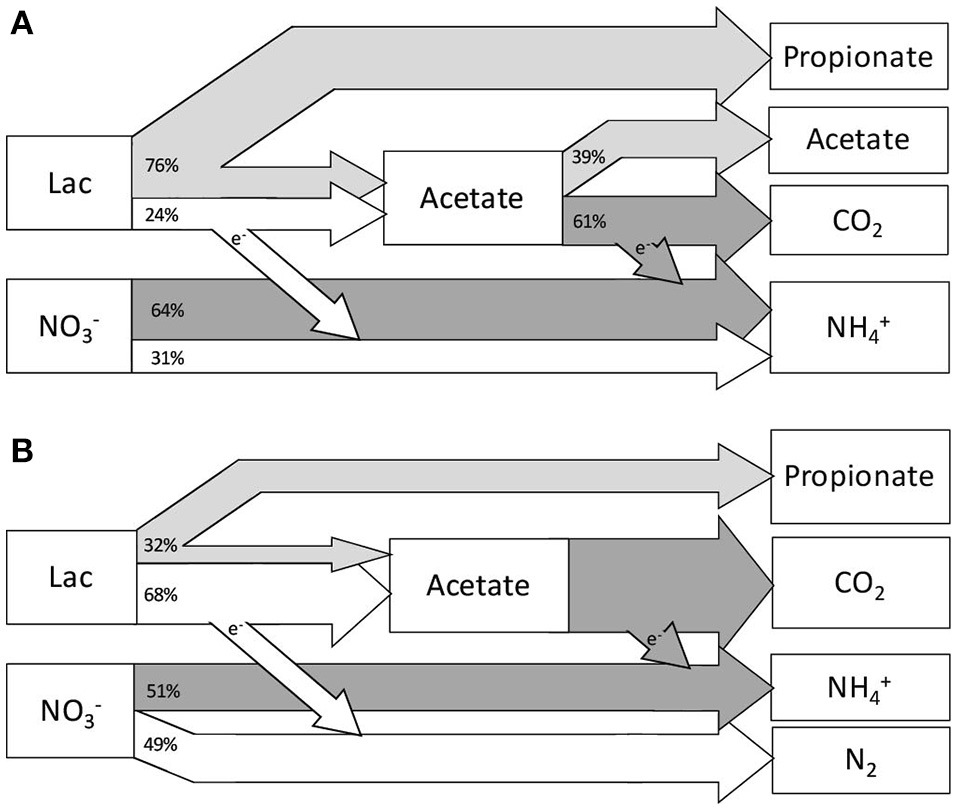
Schematic depiction of the results from the modeled pathway contributions to the steady state conversions. **(A)** Contributing conversions for the Lac/N 2.97 steady state: lactate fermentation (light gray), fermentative DNRA with partial oxidation of lactate to acetate (white) and respiratory DNRA with acetate (dark gray). Indicated is only 95% of nitrate consumption, the other 5% was assimilated in the biomass of the bacteria fermenting lactate. **(B)** Contributing conversions for the Lac/N 1.15 steady state: lactate fermentation (light gray), denitrification with partial oxidation of lactate to acetate (white), and respiratory DNRA with acetate (dark gray). Here, the nitrogen assimilated by the fermentative bacteria amounted to <1% of influent nitrate.

For the steady state of Lac/N 2.97, the nitrogen balance had a relatively small gap (8%, from Table 5A), and all nitrate was assumed to be converted into organic nitrogen in biomass and ammonium. Consequently denitrification was assumed not to play a significant role. Initially, nitrate reduction to nitrite coupled to partial oxidation of lactate to acetate was considered as a separate conversion. However, calculations demonstrated that nitrate reduction to nitrite could not account for all electrons donated by lactate oxidation. Therefore, we assumed the partial lactate oxidizing bacteria convert nitrate to ammonium. As a result, the three processes used to estimate the contribution to the overall conversions were: (i) lactate fermentation, (ii) fermentative DNRA with lactate oxidation to acetate and (iii) respiratory DNRA using acetate (Figure 3A). The estimated biomass fractions of these processes were 50% for the fermentative bacteria (of which 22% was related to fermentative DNRA), and 50% for the biomass performing respiratory DNRA with acetate.

For the steady state with influent Lac/N 1.15, i.e., still with substantial electron donor excess, nitrate was converted to both ammonia and dinitrogen gas. When respiratory DNRA was assumed to be coupled only to acetate oxidation, ammonium could be accounted for provided that partial lactate oxidation to acetate occurred. This directly implied that hypothetical partial oxidation of lactate to acetate coupled to nitrate denitrification should be included to account for removal of the remaining nitrate. No other known pathway applicable for the system could be used to obtain a correct description of the observed conversions, including pathway segregation over nitrite. Including this process resulted in a model output that described our observations adequately (Figure 3B) with a computed biomass composition of 6% lactate fermenters, 35% denitrifiers and 59% DNRA bacteria.

In the steady state receiving 0.63 Lac/N influent all lactate was converted by denitrification and concomitant assimilation, and the modeled biomass consisted fully of denitrifiers.

## Discussion

We have previously presented a mechanistic insight on the effect of the C/N ratio on the competition for nitrate between denitrification and DNRA using the non-fermentable carbon source acetate (van den Berg et al., 2016). With acetate, under nitrate limiting conditions the DNRA activity was dominant. The extent to which the factors governing the competition hold true for use of the fermentable carbon source lactate was investigated in this work. Also with lactate in great excess, Lac/N 2.97, DNRA was dominant for nitrate reduction. When the influent lactate was decreased to lactate limiting conditions, Lac/N 0.63, all lactate and nitrate were used for denitrification. Herewith the competition between DNRA and denitrification in the lactate system was comparable to the acetate experiments described previously. At the intermediate Lac/N ratio, denitrification and ammonification coexisted, but no double limitation was observed like in the former acetate study. Instead, a complex mixture of conversions was observed. A probable network of metabolic reactions is proposed on the basis of the *in silico* fit, and could be aligned with the microbial community structure observed.

### High lactate to nitrate ratio

For nitrate limiting conditions at Lac/N ratio of 2.97 mol/mol, with a substantial excess of lactate, 92% of the nitrate was converted to ammonium by DNRA, and partially used for assimilation. Since lactate was supplied in stoichiometric excess compared to nitrate, lactate not used for DNRA was expected to be used fully by fermentative bacteria. However, propionate to acetate product ratio's in combination with batch tests and molecular community analysis indicated that a more complex conversion had to occur. Batch tests performed with cells from the culture, showed that in the absence of electron acceptor lactate was fermented to acetate and propionate in a molar ratio of 1:2. This stoichiometry was also described by Seeliger et al. (2002), who had performed batch tests on pure cultures of lactate fermenting bacteria. Presumably the observed *Clostridium* and *Desulfitobacterium* species were responsible for lactate fermentation, as the observed *Geobacter* species are unlikely to consume lactate. The typical *Clostridia* members are obligate anaerobes, capable to ferment a wide range of substrates. For example, *Clostridium propionicum* is capable to ferment lactate to acetate, propionate and CO_2_, with the same stoichiometry found in our batch test experiments (Madigan and Martinko, 2005).

Acetate was not only a product of fermentation, but also an electron donor for the nitrate reduction, thereby being oxidized to CO_2_. The *Geobacter* ribotypes were the identical to the ribotypes described responsible for DNRA activity in previous studies with acetate as electron acceptor (van den Berg et al., 2015, 2016). Therefore, they were assumed to perform respiratory DNRA using acetate and not to consume lactate.

Furthermore, we observed that oxidation of propionate coupled with the reduction of nitrate to nitrite only occurred at a very low rate compared to acetate oxidation. In the chemostat culture propionate was therefore assumed not to be consumed by the nitrate reducers at significant rate and its production was used as a measure for the amount of lactate fermentation in the model evaluation. Apparently nitrate reducers oxidizing the propionate were not competitive in the system and lactate and acetate were preferred as electron donors. Using the stoichiometry for propionate production by fermentation of lactate indicated lactate use by a second process besides fermentation. Probably lactate was used directly by bacteria performing fermentative DNRA. Here, we use this term because the partial oxidation of lactate to acetate is a fermentative step (leading to substrate level ATP formation via acetylCoA), however we do not know whether or not the nitrate reduction was electrogenic. Several fermentative bacterial species have been demonstrated to perform this conversion such as *Enterobacteria* and *Clostridia* (Van Gent-Ruijters et al., 1975; Caskey and Tiedje, 1980; Bonin, 1996). In addition, species of the *Desulfitobacterium* were also able to ferment lactate and some were capable of nitrate reduction (Christiansen and Ahring, 1996; Villemur et al., 2006). Therefore, we hypothesized that the bacteria related to the *Clostridium* and *Desulfitobacterium* species are responsible for fermentative DNRA with lactate oxidation to acetate in this steady state.

Implementing these assumptions, the model based evaluation suggested a combination of three different conversions to match the overall conversions at the Lac/N ratio of 2.97 (Figure 3A); lactate fermentation, fermentative DNRA using lactate and respiratory DNRA using acetate. The bacteria performing respiratory DNRA using acetate are likely the specific *Geobacter* species, which were computed to consume 64% of the nitrate and make up 50% of the biomass, which is confirmed by the dominance of *Geobacter* identified by FISH. The fermentative bacteria were computed to consume 75% of lactate by fermentation to acetate and propionate and 25% in fermentative DNRA. These functions are assigned to the other two dominant taxa in this culture: a *Clostridium* and a *Desulfitobacterium* species. These two taxa are both capable of fermentation of lactate producing propionate and fermentative DNRA. As we cannot distinguish with the current results, we can only conclude that these two organisms were performing the two fermentative conversions. Either each performs one process, or they both perform both processes.

### Moderately high lactate to nitrate ratio

When the chemostat was operated at the decreased Lac/N ratio of 1.15, the fermentative activity decreased. Despite the supply of electron donor in adequate amounts for full nitrate reduction to ammonium, a decrease of DNRA activity was observed. Only 61% of nitrate was reduced to ammonium by DNRA or used for assimilation. The remaining nitrate was reduced to dinitrogen gas by denitrifiers, as their presence was indicated in batch tests performed with the culture enriched at Lac/N ratio of 1.15 and acetylene. With the assumption that respiratory DNRA occurred only via acetate oxidation in our system, we could not find a system based on reported pathways for anaerobic/anoxic lactate oxidation which could describe our data. Therefore, we hypothesized that partial lactate oxidation to acetate coupled to denitrification occurred in our enrichment culture. This proposed process was presumably performed by the ribotype related to *P. militaris* strain MP, which was dominant in the culture at Lac/N ratio 1.15 next to the previous found *Clostridales* bacteria and *Geobacter* species. This *Betaproteobacterium* has been described as a non-fermentative, strictly respiring facultative anaerobe capable of nitrate and nitrite denitrification with acetate, propionate or lactate (Thrash et al., 2010). Therefore it was presumed to perform the denitrification with partial oxidation of lactate in the culture.

Assuming that no denitrification using acetate occurs, in the steady state culture receiving 1.15 Lac/N the three parallel processes modeled were lactate fermentation to acetate and propionate, denitrification using lactate oxidation to acetate, and DNRA using acetate. Fermentation of lactate and DNRA using acetate were again attributed to the *Clostridiales* and *Geobacter* members respectively. The denitrifiers were presumed to relate to the *P. militaris* ribotype. With this interpretation, the model suggests that half of the nitrate is denitrified and the denitrifying *Betaproteobacterium* makes up 35% of the total population. The acetate-using ammonifying *Geobacter* species would make up 59% of the population, and the fermenting bacteria 6%, which is confirmed by the dominance of *Geobacter* identified by FISH.

In the steady state receiving influent Lac/N ratio of 0.63 mol/mol, a dual limitation of electron donor and acceptor was expected. All influent nitrate and lactate were used in denitrification and the DNRA and fermentative bacteria were outcompeted. However, in the community next to the dominant denitrifier, related to the *P. militaris* ribotype, also the *Geobacter* was present. For a similar steady state of dual limitation with acetate and nitrate, where only denitrification seemed to occur, the presumed DNRA bacterium was also present (van den Berg et al., 2016). It was speculated that this organism might produce less ammonia than the denitrifiers consume, and that as a result no residual ammonia had been detected. Adapting the model for denitrifiers to use ammonium for growth showed that the DNRA biomass could amount up to 15%, without net ammonia production in the culture.

### Nature of the carbon source

The overall results suggest that use of a fermentable carbon source affects the competition for nitrate between DNRA and denitrification compared to a non-fermentable source. For the non-fermentable substrate acetate, when provided in excess, the nitrate was reduced to ammonium (van den Berg et al., 2016). In contrast, for supply of lactate at a comparable amount of electron equivalents (at Lac/N ratio 1.15, comparable to Ac/N 1.87, Table 1) a lower DNRA activity of 58% of the nitrate reduction was observed, even though nitrate was limiting and the electron donor lactate was provided in excess. Only for high excess of lactate, at the Lac/N ratio of 2.97, all nitrate was converted to ammonia. Qualitatively, the decrease of DNRA activity with the decrease of Lac/N ratio is similar for acetate and lactate, and was also observed for the fermentable substrate glucose (Akunna et al., 1994). However, for acetate a direct mechanistic coupling of conversions at a certain Ac/N to metabolic Ac/N stoichiometries was derived, which does not apply in the case of lactate. Possibly the lactate consumption by the fermenters limited electron donor availability for nitrate reduction. Fermenters are fast consumers and growers and could therefore have a sufficiently high affinity (μ_max_/K_S_) for lactate to be competitive with the dissimilatory processes, despite their lower ATP yield per lactate converted (Kraft et al., 2011). Hence, fermentative lactate consumption creates a dual substrate limitation for the nitrate reducers. Just as for acetate grown enrichments, the dual limitation at lower Lac/N ratio also resulted in coexistence of denitrification with DNRA. However, the fermentative bacteria are only competitive for the energy source to a certain extent, because they were outcompeted by the denitrifiers in the steady state at Lac/N 0.63, where both lactate and nitrate influent concentrations were limiting. It is remarkable to see that at the intermediate Lac/N ratio denitrification and DNRA coexisted and both lactate and acetate were limiting. Only propionate remained in the reactor effluent. Apparently, the type of organic carbon limiting the conversion has an impact on the nitrate reduction pathway obtained. It remains unclear why acetate limitation as obtained at Lac/N = 1.15 favors lactate oxidation to acetate coupled to denitrification, and acetate excess favors fermentative DNRA.

Studies in the environment or with environmental slurries regard the C-source mostly as labile carbon forms and non-labile forms, of which the latter are harder to degrade than the former (Giblin et al., 2013; Plummer et al., 2015). In some studies different labile energy sources are compared, e.g., Morley and Baggs (2010) described 4% of nitrate converted to ammonium for the fermentable glucose, the highest formation in their studies. Other studies on the impact of the nature of the carbon source focused on nitrate removal efficiency in wastewater treatment systems. Batch test have been reported with e.g., biofilms, aerobic and anaerobic granular sludge comparing substrates as glucose and acetate. Generally, glucose and acetate showed similar efficiency for removal, only for glucose some nitrite and/or ammonia accumulation is observed (<4%) (Her and Huang, 1995; Srinandan et al., 2012; Chen et al., 2016). The result of this study establishes the difference in the competition for nitrate between DNRA and denitrification for use of lactate, compared to acetate. The presence of fermentative bacteria, in addition to the nitrate reducers, increases the range of apparent available substrate C/N ratios, for which the denitrifiers and DNRA bacteria coexist. As a consequence, the amount of DNRA activity expected based on electron donor availability, as suggested by van den Berg et al. (2016), is probably lower in practice when (part of) the available electron donors are fermentable. As it implies higher nitrate removal through denitrification, this is a positive result for wastewater treatment.

## Conclusion

In this study we showed that the C/N effect on the nitrate competition between DNRA and denitrification in enrichment chemostat cultures for acetate is qualitatively similar for lactate as electron donor. However, the coupling of the range of dual substrate limitation to the process Ac/N stoichiometry cannot readily be extrapolated. Apparently, fermentative bacteria are competitive for lactate and can thereby limit the availability for the preferred carbon source(s) for the obligate nitrate reducing bacteria. The altered ratio of apparent substrates available affects the competition between denitrifiers and DNRA bacteria for nitrate in favor of the denitrification. Furthermore, for the obtained steady states we were able to identify the pathways likely responsible for the overall system function and couple this to the community structure. In the steady state receiving influent Lac/N of 2.67, three processes co-occurred: fermentation of lactate to acetate and propionate and fermentative DNRA, performed by two species of *Clostridia*, and respiratory DNRA using acetate, performed by two *Geobacter* species. For the Lac/N 1.15 mol/mol steady state, fermentation and DNRA, coupled to the same taxa, had decreased and denitrification played a significant role in the conversions, which was presumably linked to the presence of the Betaproteobacterium related to *P. militaris*. The results improve our understanding for the C/N effect on the competition between nitrate reducers and helps predict DNRA or denitrification contributions in aqueous environments, e.g., wetlands or wastewater treatment systems.

## Author contributions

EB wrote the manuscript and performed parts of lab work. ME performed most lab work. All authors made substantial contributions to the experimental design, interpretation of the results and revisions of the manuscript.

### Conflict of interest statement

The authors declare that the research was conducted in the absence of any commercial or financial relationships that could be construed as a potential conflict of interest.
