# Supplementary material for: Fermentative Bacteria Influence the Competition between Denitrifiers and DNRA Bacteria
Source: Front Microbiol. 2017 Sep 5;8:1684. doi: 10.3389/fmicb.2017.01684 (PMC5591879; doi:10.3389/fmicb.2017.01684)
Supplement: Supplementary file 1 [file DataSheet1.PDF]

Supplementary Material

Fermentative bacteria influence the competition between dentirifiers and DNRA bacteria

Eveline M. van den Berg[1,*], Marina Elisário[1], J. Gijs Kuenen[1], Robbert Kleerebezem[1] and Mark C. M. van Loosdrecht[1]

[1]Environmental Biotechnology group, Department of Biotechnology, Delft University of Technology, Delft, The Netherlands

*** Correspondence:** Eveline van den Berg: E.M.vandenBerg@tudelft.nl

1 Supplementary Tables

Table S1. Acid/base equilibrium and respective pKa considered in calculation of the bicarbonate concentration in the chemostat for the different steady states. Equilibrium between H_3PO_4 and $H_2PO_4^-$ was not taken into consideration since the correspondent pka (equal to 2.3) is so low comparing with the working pH. T, the electro-neutrality equation for the charged species in the chemostat solved was written as $[H^+] + [Na^+] + [K^+] = [HCO_3^-] + 2.[CO_3^{2-}] + [OH^-] + [Ac^-] + [Prop^-] + [Cl^-] + [H_2PO_4^-] + 2.[HPO_4^{2-}] + 3.[PO_4^{3-}]$.

Equilibria	pKa
$H_2CO_3^* \rightleftarrows HCO_3^- + H^+$	6.37
$HCO_3^- \rightleftarrows CO_3^{2-} + H^+$	10.36
$HAc \rightleftarrows Ac^- + H^+$	4.76
$HProp \rightleftarrows Prop^- + H^+$	4.88
$H_2PO_4^- \rightleftarrows HPO_4^{2-} + H^+$	7.21
$HPO_4^{2-} \rightleftarrows PO_4^{3-} + H^+$	12.32
$NH_4^+ \rightleftarrows NH_3 + H^+$	9.26

Table S2. Sequences generated in the amplicon sequencing, 250bp paired-end raw reads (Raw PE), and read numbers in subsequent processing steps.

Sample	Raw PE(#)	Combined(#)	Qualified(#)	Nochime(#)	AvgLen(nt)	Effective%
2.97a	73,325	69,096	61,146	59,936	429	81.74
2.97b	74,349	69,926	61,762	60,477	427	81.34
1.15a	62,44	58,809	52,202	51,293	429	82.15
1.15b	71,999	67,506	60,012	59,021	429	81.97
0.63	76,412	71,041	62,192	61,58	428	80.59

Table S3. Alpha diversities calculated for the different samples analyzed by amplicon sequencing. In the calculations normalized OTU abundances, which were normalized using a standard of sequence number corresponding to the sample with the least sequences, were used.

Sample	Observed species	Shannon	Simpson	Chao1	ACE	Goods coverage
2.97a	333	2.7	0.7	423	447	0.998
2.97b	281	2.5	0.6	347	364	0.998
1.15a	317	2.3	0.7	403	420	0.998
1.15b	272	1.7	0.5	317	344	0.998
0.63	353	1.5	0.4	454	475	0.997

Table S4a Test with SILVA TestProbe (database SSU 128, sequence collection REFNR) for FISH probe GeobacII_464.

Mismatches allowed	Matches	
0	0	-
1	0	-
2	1	uncultured in genus *Geobacter*

Table S4b Test with RDP ProbeMatch for FISH probe GeobacII_464.

Mismatches allowed	Matches	
0	0	-
1	3	uncultured in genus *Geobacter*
2	5	uncultured in genus *Geobacter* (4)<br>uncultured in family *Desulfobacteraceae* (1)

Table S5. Conversion rates of the different substrates and products (mM/h) observed in the batch tests performed with the steady state biomass from culture receiving Lac/N ratio 2.97 mol/mol. Rates were calculated by linear regression of the different concentrations over time and respective standard deviations by the function *LINEST* in *Microsoft Office Excel*.

Substrates	reference to fig S1	Conversion rates ($mmol.h^{-1}.L^{-1}$)				N% to NH_4^+
		Lac.	Ac. [a)]		Prop.	
Lac.	A	-3.02 ± 0.35	1.01 ± 0.15	-	1.87 ± 0.27	0
Lac. + NO_3^-	B	-2.46 ± 0.34	0.58 ± 0.15	-0.52 ± 0.00	1.08 ± 0.15	83.5
Lac. + NO_2^-	C	-2.12 ± 0.22	0.16 ± 0.15	-0.15 ± 0.00	1.09 ± 0.10	85.5
Ac. + NO_3^-	D	-	-	-0.97 ± 0.07	-	86.0
Ac. + NO_2^-	E	-	-	-0.82 ± 0.14	-	84.9
Prop. + NO_3^-	F	-	-	-	-0.14 ± 0.04	10.8
Prop. + NO_2^-	G	-	-	-	-0.01 ± 0.13	7.5

[a)] When acetate was first produced and later used as a substrate, positive and negative rates are presented corresponding to production and consumption, respectively.

Table S6. Conversion rates of the different substrates and products (mM/h) observed in the batch tests performed with the steady state biomass from culture receiving Lac/N ratio 1.15 mol/mol. Rates were calculated by linear regression of the different concentrations over time and respective standard deviations by the function *LINEST* in *Microsoft Office Excel*.

Substrates	reference	Conversion rates ($mmol.h^{-1}.L^{-1}$)			N-conversion end products (N%)		
	to fig S2	Lac.	Ac.	Prop.	NH_4^+	N_2O	to N_2 [a)]
Lac.	A	-1.50 ± 0.07	0.50 ± 0.01	0.98 ± 0.03	-	-	n.d.
Lac. + NO_3^-	B	-1.55 ± 0.04	0.51 ± 0.00	0.90 ± 0.19	2	0.4	77
Lac. + NO_2^-	C	-0.68 ± 0.08	0.26 ± 0.00	0.13 ± 0.00	3	4	n.d.
Ac. + NO_3^-	D	-	-1.28 ± 0.11	-	2	-	84
Ac. + NO_2^-	E	-	-0.90 ± 0.12	-	10	7	n.d.
Prop. + NO_3^-	F	-	-	-1.51 ± 0.04	3	4	56
Prop. + NO_2^-	G	-	-	-0.01 ± 0.02	3	-	n.d.

a) Percentage estimated based on measurements of N_2O fraction in the headspace of the additional batch vials, which were inoculated with 5 %(v/v) acetylene.

n.d. Not determined.

2 Supplementary Figures

2.1 Supplementary Figure 1

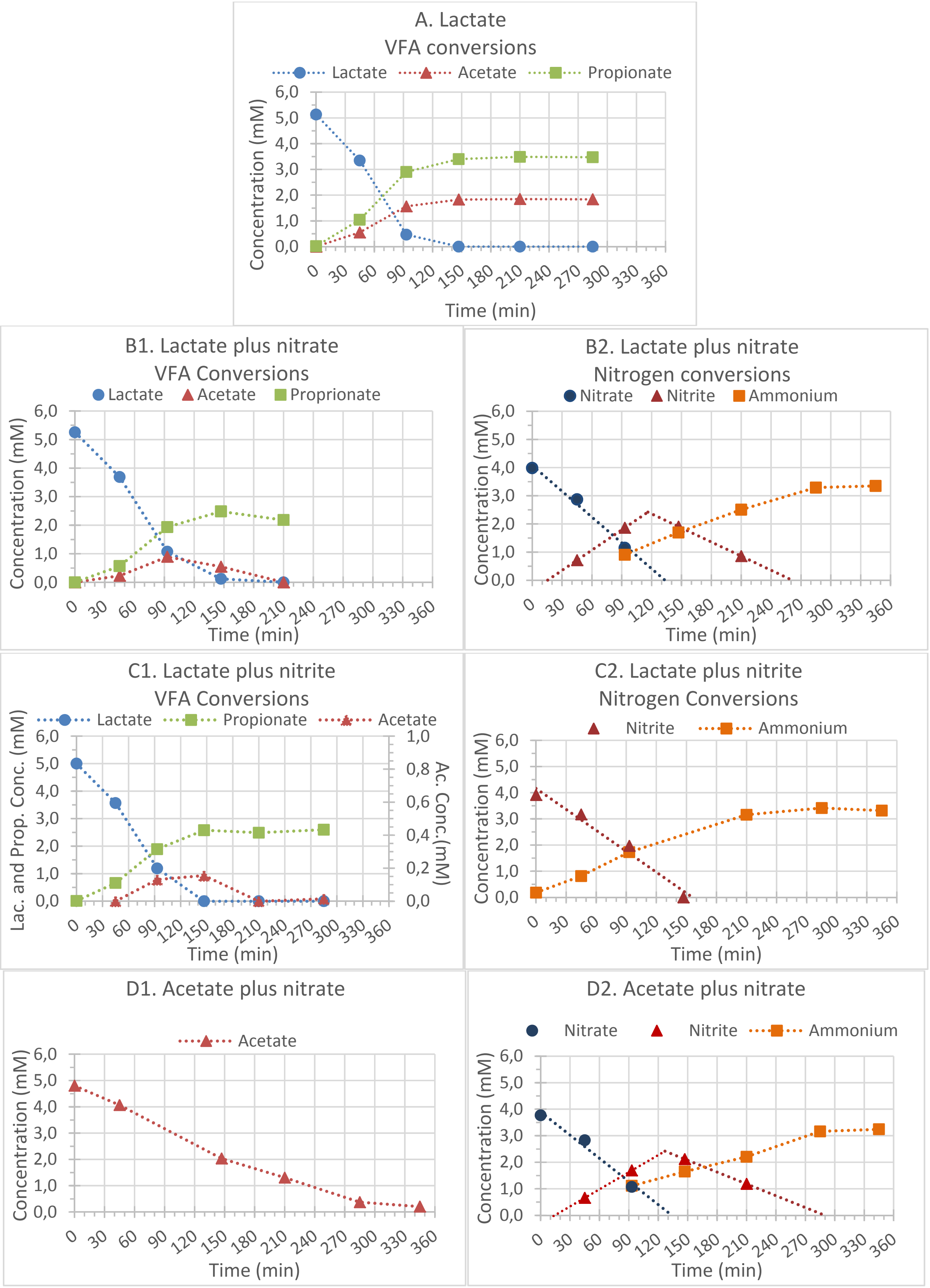

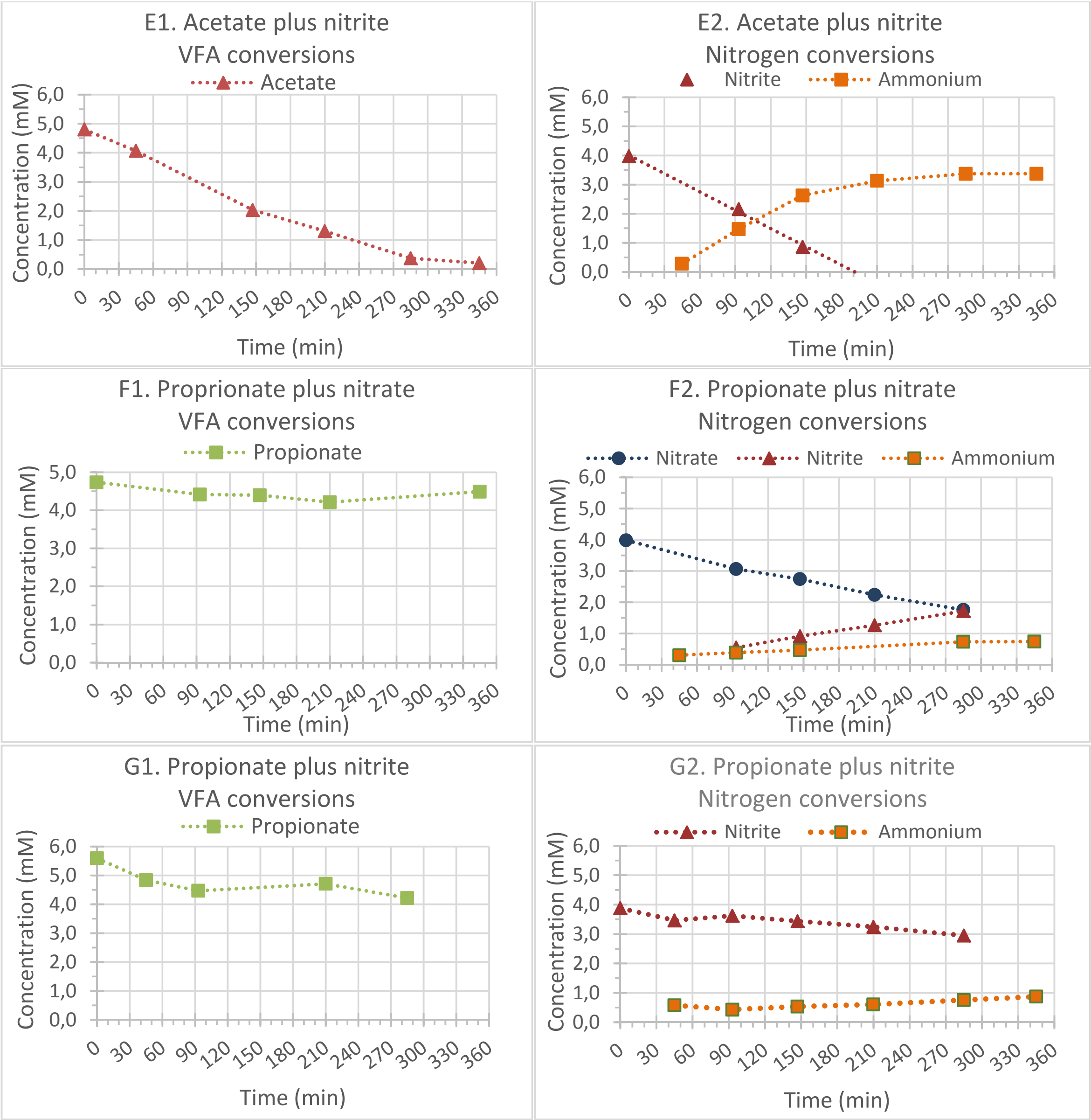

Figure S1. Concentration profiles of the simultaneous batch tests performed with the steady state biomass from culture receiving Lac/N ratio 2.97 mol/mol. Initial electron donor concentrations were 5 mM of and electron acceptor concentrations were 4 mM. Note that transient accumulation of nitrite occurred when nitrate was the electron acceptor with either lactate or acetate as carbon source.

2.2 Supplementary Figure 2

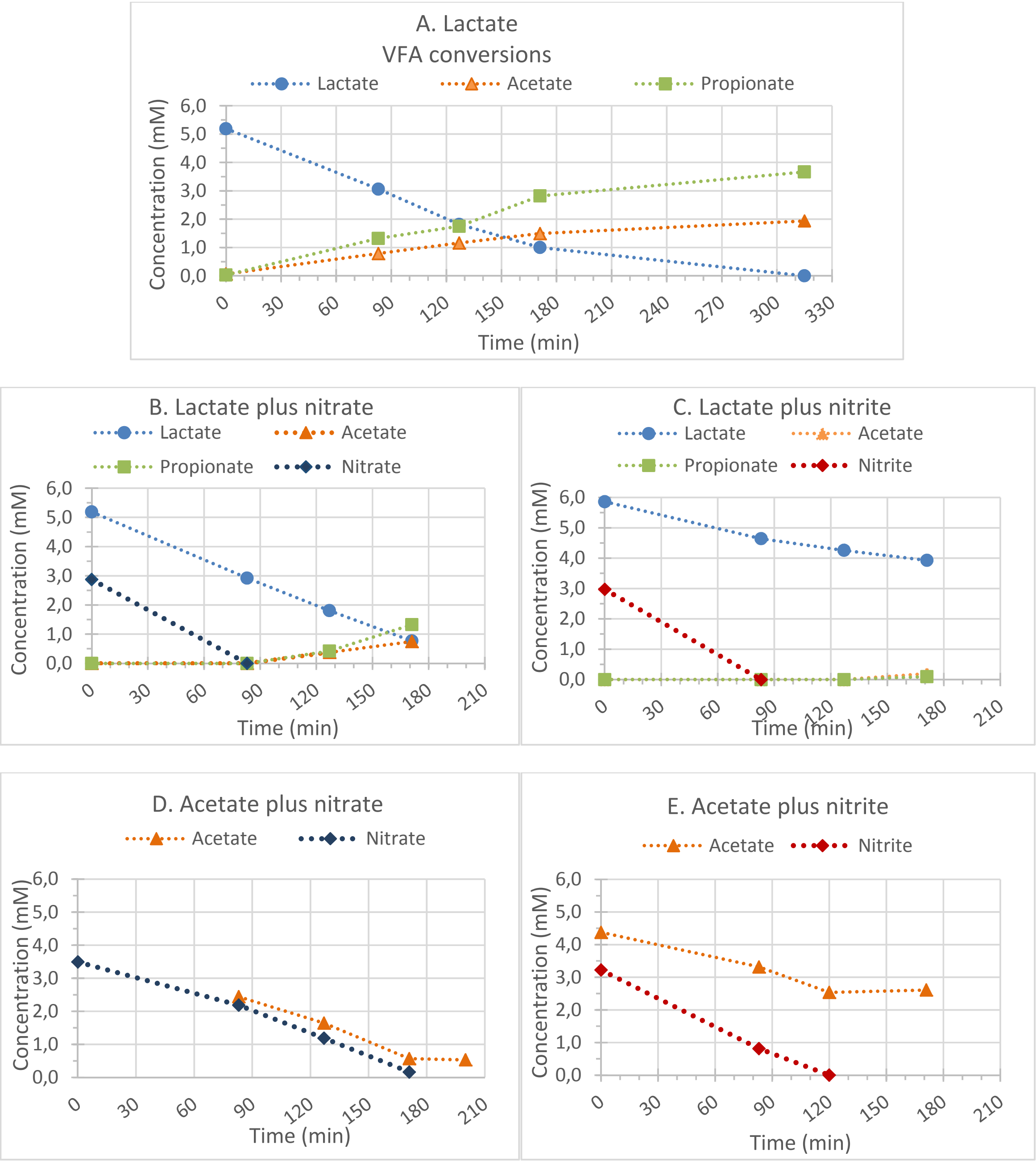

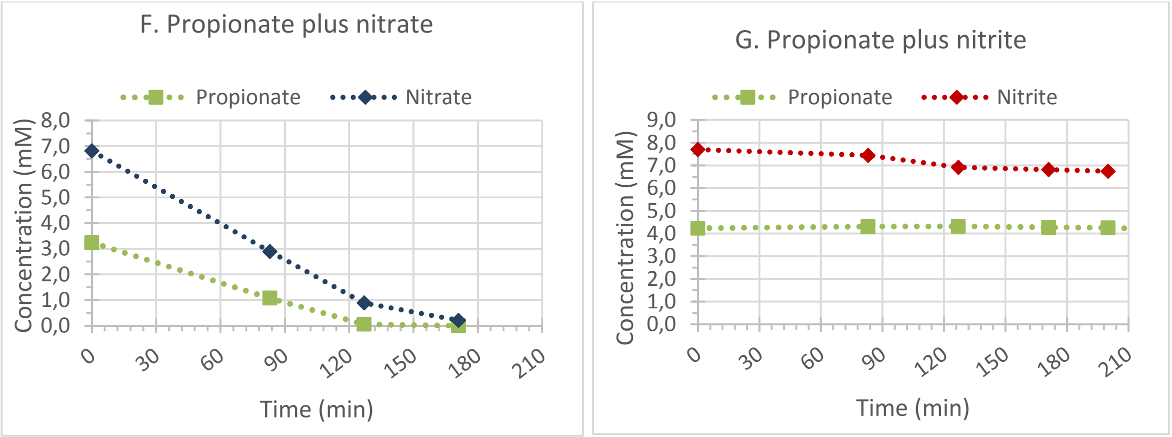

Figure S2. Concentration profiles of the simultaneous batch tests performed with the steady state biomass from culture receiving Lac/N ratio 1.15 mol/mol. Initial electron donor concentrations were 5 mM of and electron acceptor concentrations were 4 mM.

2.3 Supplementary Figure 3

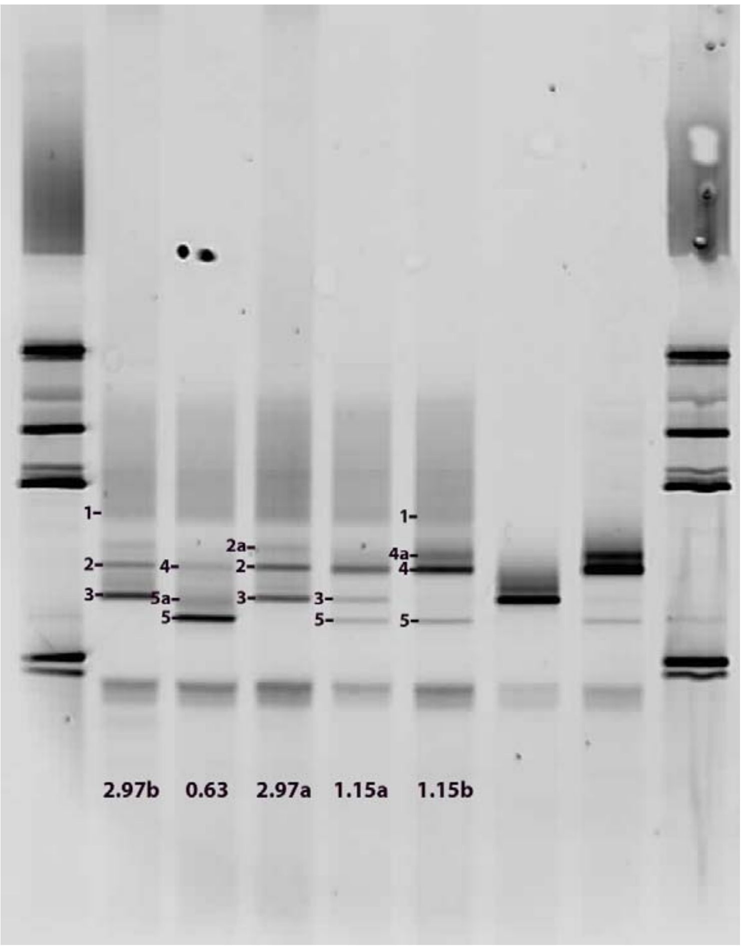

Figure S3. DGGE gel picture. The same DNRA extracts were analyzed as in the amplicon sequencing (figure 2) and the sample names in the lane are the same as in the amplicon result. The other lanes belong to other research. Bands labeled with the same number contained the same sequence, and the sublabel 'a' was given to slightly different sequences which related to the same species. BLASTn result for closest related species and identities: band 1 *Clostridium* sp. SW001 (99%); band 2 *Desulfitobacterium hafniense* (99%); band 3 *Geobacter luticola* (97%); band 4 *G. lovleyi* (97%); band 5 *Propionivibrio militaris* (99%).
